# Oral Delivered Dexmedetomidine Promotes and Consolidates Non-rapid Eye Movement Sleep via Sleep–Wake Regulation Systems in Mice

**DOI:** 10.3389/fphar.2018.01196

**Published:** 2018-12-05

**Authors:** Zhen-Xin Feng, Hui Dong, Wei-Min Qu, Wei Zhang

**Affiliations:** ^1^Department of Anesthesiology, The First Affiliated Hospital of Zhengzhou University, Zhengzhou, China; ^2^Department of Pharmacology, School of Basic Medical Sciences, Fudan University, Shanghai, China

**Keywords:** dexmedetomidine, LMA, EEG/EMG, sleep–wake, c-Fos

## Abstract

Dexmedetomidine, a highly selective α2-adrenergic agonist, is widely used in clinical anesthesia and ICU sedation. Recent studies have found that dexmedetomidine-induced sedation resembles the recovery sleep that follows sleep deprivation, but whether orally delivered dexmedetomidine can be a candidate for the treatment of insomnia remains unclear. In this study, we estimated the sedative effects of orally delivered dexmedetomidine by spontaneous locomotor activity (LMA), and then evaluated the hypnotic effects of dexmedetomidine on sleep–wake profiles during the dark and light phase using electroencephalography/electromyogram (EEG/EMG), respectively. Using c-Fos staining, we explored the effects of dexmedetomidine on the cerebral cortex and the sub-cortical sleep–wake regulation systems. The results showed that orally delivered dexmedetomidine at 2 h into the dark cycle reduced LMA and wakefulness in a dose-dependent manner, which was consistent with the increase in non-rapid eye movement sleep (NREM sleep). However, dexmedetomidine also induced a rebound in LMA, wake and rapid eye movement sleep (REM sleep) in the later stage. In addition, orally delivered dexmedetomidine 100 μg/kg at 2 h into the light cycle shortened the latency to NREM sleep and increased the duration of NREM sleep for 6 h, while decreased REM sleep for 6 h. Sleep architecture analysis showed that dexmedetomidine stabilized the sleep structure during the light phase by decreasing sleep–wake transition and increasing long-term NREM sleep (durations of 1024–2024 s and >2024 s) while reducing short-term wakefulness (duration of 4–16 s). Unlike the classic hypnotic diazepam, dexmedetomidine also increased the delta power in the EEG spectra of NREM sleep, especially at the frequency of 1.75–3.25 Hz, while ranges of 0.5–1.0 Hz were decreased. Immunohistochemical analysis showed that orally delivered dexmedetomidine 100 μg/kg at 2 h into the dark cycle decreased c-Fos expression in the cerebral cortex and sub-cortical arousal systems, while it increased c-Fos expression in the neurons of the ventrolateral preoptic nucleus. These results indicate that orally delivered dexmedetomidine can induce sedative and hypnotic effects by exciting the sleep-promoting nucleus and inhibiting the wake-promoting areas.

## Introduction

Sleep exists in the overwhelming majority of organisms, from humans to worms (Allada and Siegel, 2008). Sleep has an essential role in many vital physiologic functions, including energy conservation, brain waste clearance, cognition, memory, and modulation of the immune system (Zielinski et al., 2016). Insomnia is a common sleep disorder, defined as a complaint of prolonged sleep latency, difficulties in maintaining sleep, and subsequent impairments in daytime functioning (Riemann et al., 2010). It has been identified as a critical and growing cause of concern for public health (Nomura et al., 2010).

Treatment for insomnia is still mainly drug-based therapy. The pharmacologic approach primarily includes benzodiazepines and non-benzodiazepines. These classic hypnotics all act on gamma-aminobutyric acid type A receptors, mediating inhibition of the CNS and improving sleep related problems (Richey and Krystal, 2011; Uygun et al., 2016). However, these drugs reduce the depth of NREM sleep, and thus do not mimic physiological sleep (Akeju and Brown, 2017). In addition, these “sleeping pills” are associated with neurocognitive dysfunction (Manconi et al., 2017; de Zambotti et al., 2018). Other hypnotics, such as antihistamines, antipsychotics, and melatonin, are not recommended for insomnia treatment due to side-effects according to the European Guideline for the diagnosis and treatment of insomnia (Riemann et al., 2017).

Accumulating experimental evidence indicates that anesthesia and sedatives to a lesser or greater extent exert their function in natural sleep-wake circuits (Franks, 2008; Han et al., 2014). Dexmedetomidine, a α2-adrenoceptor agonist, has been widely used in clinical anesthesia and ICU sedation (Mantz et al., 2011). Patients receiving dexmedetomidine for effective sedation are still easily aroused from a state similar to sleep that is not observed in other clinical anesthetics (Venn et al., 1999; Huupponen et al., 2008). According to a previous study, the locus coeruleus (LC) is pivotal in inducing the hypnotic response to dexmedetomidine (Correa-Sales et al., 1992). Electrophysiology studies confirm that dexmedetomidine opens inwardly, rectifying potassium channels to hyperpolarize the discharge of LC neurons and reduce norepinephrine (NA) release (Chiu et al., 1995). Decreased NA levels release the inhibition of the preoptic area, resulting in excitement of the sleep-promoting nucleus, thereby inhibiting the wake-promoting areas through mediating gamma-aminobutyric acid (GABA) and galanin (Nelson et al., 2003; Zhang et al., 2015). These advances suggest that dexmedetomidine may converge on the endogenous sleep pathway to introduce sedation.

Although dexmedetomidine induced sedation resembles the recovery sleep that follows sleep deprivation (Zhang et al., 2015), it remains unclear whether it could be an ideal candidate for the treatment of insomnia. An ideal agent should effectively shorten sleep latency, increase the amount of sleep time, stabilize the sleep structure, and insure sleep depth. In addition, convenient routes of administration, such as oral delivery, can improve the compliance of patients. Intravenous administration is not only inconvenient to self-operate, but it also has the risk of bacterial infection and pain. However, in clinical practice, dexmedetomidine is always given as a continuous intravenous pump infusion (Ohtani et al., 2011) or nasal drip in pediatric sedation (Li et al., 2016). In preclinical research, intraventricular injection or intraperitoneal administration is the most common route of administration. Until now, the sedative and hypnotic effects of orally delivered dexmedetomidine remain unclear and the impacts of dexmedetomidine on sleep structure are not yet fully understood. Moreover, dexmedetomidine can still induce hypnotic effect in mice unable to synthesize NA (Gilsbach et al., 2009; Hu et al., 2012; Sanders and Maze, 2012; Garrity et al., 2015) or with selective knockdown of alpha-2A adrenergic receptors in the LC (Zhang et al., 2015), suggesting that dexmedetomidine-induced hypnosis may depend on other brain areas in addition to the LC.

Here, we used LMA and EEG/EMG to investigate whether orally delivered dexmedetomidine has sedative and hypnotic effects during the dark phase when mice are active. We then, evaluated the effects of dexmedetomidine on the time of NREM sleep, NREM sleep latency, number of sleep-wake transitions, structure of sleep-wake profiles, and the delta power density of NREM sleep during the light phase when mice were sleepy. Finally, the effects of dexmedetomidine on the expression of c-Fos protein in the brain were explored by immunohistochemistry.

## Materials and Methods

### Animals

Male SPF C57BL/6J mice aged 8–10 weeks (weighing 20–24 g) were purchased from the Laboratory Animal Center, Chinese Academy of Sciences (Shanghai, China). The mice were housed in an insulated and soundproofed room maintained at an ambient temperature of 22 ± 0.5°C with a relative humidity of 60 ± 2% under an automatically controlled 12-h light/12-h dark (L/D) cycle [light on at 7:00 A.M, illumination intensity≈10 lx(Zhang et al., 2016)]. Food and water were available *ad libitum*. Experimental protocols were approved by the Medical Experimental Animal Administrative Committee of Shanghai and strictly followed the Guidelines from the National Institute of Health (U.S.) regarding the care and use of animals for experimental procedures. Every effort was made to minimize the number of animals for experiments and any pain or discomfort they experienced.

### Chemicals

Dexmedetomidine was obtained from the Jiangsu HengRui Pharmaceutical Co. Ltd. (Jiangsu, China). Rabbit polyclonal anti-c-Fos antibody was purchased from Abcam (Cambridge, MA, United States). Biotinylated donkey anti-rabbit IgG and avidin-biotin-peroxidase were purchased from Vector Laboratories (Burlingame, CA, United States). Finally, 3, 3-diaminobenzidine-tetra-hydrochloride (DAB) was purchased from Sigma-Aldrich (St. Louis, MO, United States). Dexmedetomidine was dissolved in sterile saline before use.

### Spontaneous Locomotor Activity in an Accustomed Environment

Spontaneous locomotor activity was measured according to the method described previously (Inoue et al., 1996). The recording system mainly consisted of soundproof cabinets, a transparent recording cage (L 280 mm, W 230 mm, H 210 mm), a continuous infrared detector, recording software, and a monitor (Biotex, Kyoto). The bottom of each recording cage was divided into an average of 256 unit areas. Mouse movements in each unit area were identified by the infrared detector, and recorded as one activity. Activity was automatically monitored and calculated every 5 min. Before the start of recording, mice were housed individually in a transparent recording cage with food and water available, and habituated over three consecutive days. On the experimental day, the control group received saline while treatment groups received dexmedetomidine.

### Polygraphic Recordings and Vigilance State Analysis

Under pentobarbital anesthesia at 50 mg/kg (i.p.), mice were chronically implanted with EEG/EMG electrodes for polysomnographic recordings (Huang et al., 2005). The implant consisted of two stainless-steel screws (1 mm in diameter) and EEG electrodes inserted through the skull of the cortex (+1.0 mm anteroposterior; -1.5 mm mediolateral from the bregma or lambda) according to the atlas of Paxinos and Franklin (2013) and served as EEG electrodes. Two insulated stainless steel wires were bilaterally placed into both trapezius muscles and served as EMG electrodes. All of the electrodes were linked to a mini-connector and fixed to the skull with dental cement. After a 7-day recovery period, the mice were housed individually in transparent barrels and habituated to the recording cable for 7 days before recording was started. The uninterrupted synchronous recordings of EEG and EMG were performed by means of a slip ring, which was designed for letting the mice move freely.

As previously described (Huang et al., 2005), cortical EEG and EMG signals were amplified and filtered (EEG, 0.5–30Hz; EMG, 20–200Hz) and then digitized at a sampling rate of 128 Hz and recorded with *SleepSign* (Kissei Comtec, Nagano, Japan). After the experiment was completed, the EEG/EMG data were automatically classified off-line using 4 s epochs for wakefulness, REM sleep, and NREM sleep using *SleepSign* software according to standard criteria. These automatically defined classifications were checked manually and corrected if necessary.

EEG power spectra were calculated through fast Fourier transform at the frequency range 0–25 Hz, with a resolution of 0.25 Hz (Qu et al., 2010), and relative power bands were summed as: delta, 0.5–4 Hz; theta, 6–10 Hz; alpha, 12–14 Hz; and beta, 15–25 Hz. Every 4 s epochs of EEG power spectra were calculated through FFT. State-dependent spectral power was averaged by pick corresponding state epoch in a state-dependent manner. The power spectra was normalized by calculation of the percentage of each 0.25 Hz bin from the total power of each individual animal. The power of each 0.25 Hz bin was first averaged for each specific stage (NREM sleep, REM sleep, Wake) individually, and then normalized as a group by calculation of the percentage of each bin from the total power (0–24.75 Hz) of the individual animal.

### Pharmacological Treatments

Dexmedetomidine was dissolved in sterile saline immediately before use and implemented by intragastrical administration (i.g.) in a volume of approximately 10 ml/kg at doses of 25, 50, 100, and 200 μg/kg. To study the sedative effect of dexmedetomidine in mice, spontaneous locomotor activity was tested consecutively for 24 h from 19:00. The mice received saline or dexmedetomidine at 21:00.

To estimate possible drowsiness or hypnotic effects resulting from dexmedetomidine in the dark phase for mice, EEG/EMG was tested consecutively for 48 h from 19:00. On the first day of the experiment, all of the groups of mice received saline at 21:00 (in the early phase of the dark period), and the recordings made on that day served as self-controls. On the second day, the same mice were administered with (i.g.) saline or dexmedetomidine (25, 50, 100, and 200 μg/kg,) at the same time, and the recordings made on the second day served as the experimental data.

To evaluate the effects of oral delivery dexmedetomidine on sleep-wake regulation in mice during the light phase (sleep stage for mice), EEG/EMG was tested consecutively for 48 h from 07:00. At the same time, we compared the route of i.g. with intraperitoneal injection (i.p.) dexmedetomidine, and diazepam was the positive control drug. On the first day of the experiment, mice received saline at 09:00 (in the early phase of the light period), and the recordings made on that day served as self-controls. On the second day, the same mice were administered with dexmedetomidine (100 μg/kg i.g. or i.p.) or diazepam (6 mg/kg, i.g.) at the same time, and the recordings made on the second day served as the experimental data.

### c-Fos Immunochemistry and Cell Counting

In order to evaluate the effects of dexmedetomidine on c-Fos expression in the cerebral cortex and sleep–wake control pathway, animals were divided into two groups. Each group was given either saline or dexmedetomidine 100 μg/kg i.g. at 21:00, and animals were then sacrificed after 120 min for immunohistochemical experiments as described previously (Qiu et al., 2014). The mice were anesthetized using 5% chloral hydrate (500 mg/kg) and perfused with saline solution followed by 4% paraformaldehyde (PFA) in 0.1 M phosphate buffer (PB, pH 7.0) through the heart. The brains were immediately removed and post-fixed in 4% PFA in 0.1 M PB (pH 7.4) for 4 h. The brains were then transferred to 20% sucrose in phosphate-buffered saline (PBS) and kept in the solution until they sank to the bottom. Frozen sections were cut at 30 μm in coronal planes by using freezing microtome (Jung Histocut, model 820-II, Leica, Nussloch, Germany). Sections were washed in 0.01 M PBS and then incubation with c-Fos antibody. The antibody was diluted 1:10000 in antiserum solution 2 (1% normal BSA, 0.2% Triton X-100, and 0.4% sodium azide in 0.01 M PBS at pH 7.2) at room temperature overnight. On the second day, the sections were incubated with a 1:1000 dilution of biotinylated goat anti-rabbit secondary antibodies for 2 h followed by a 1:1000 dilution of avidin–biotin–peroxidase for 1 h at 37°C. The peroxidase reaction was visualized with 0.05% DAB in 0.1 M phosphate buffer and 0.01% H_2_O_2_. Sections were mounted, dehydrated, and cover slipped. The sections were then examined under bright-field illumination using a Leica DMLB2 microscope (Leica Microsystems, Wetzlar, Germany). Images were captured by a Cool SNAP-Proof digital camera (SPOT RTKE Diagnostic Instruments, Sterling Heights, MI, United States). For the cerebral cortex, the 200 μm × 200 μm counting box was placed in the center of cortex of three adjacent sections, and only black-stained large neurons (likely pyramidal neurons) counted as c-Fos positive neurons. For the ventrolateral preoptic nucleus (VLPO), lateral hypothalamus (LH), tuberomammillary nucleus (TMN), laterodorsal tegmental nucleus (LDT), medial parabrachial nucleus (MPB), lateral parabrachial nucleus (LPB), and LC, we counted all of the c-Fos positive neurons in the entire region of three adjacent sections. The c-Fos counts were represented by average counts per section and per side.

### Statistical Analysis

All data subjected to statistical analysis in SPSS 19.0. All of the results are expressed as means ± SEM. For the time course data, the hourly amounts of LMA and each sleep–wake stage profiles in mice treated with saline or dexmedetomidine were compared using two-way ANOVA followed by Fisher’s least-significant difference test. Histograms of the amounts of LMA, sleep, and wakefulness were assessed by one-way ANOVA followed by Bonferroni tests. Histograms of sleep latency were analyzed using the two-tailed paired *t*-tests, with each animal serving as its own control. Comparisons of sleep counts, as well as the number of sleep/wake events, duration, and transition, and the number of c-Fos immunoreactivity neurons were performed using unpaired, two-tailed Student’s *t-*tests. Graphs of the power density of NREM sleep and the quantitative changes in power for the delta (0.5–4.0 Hz) frequency bands were analyzed using the two-tailed paired Student’s *t*-test. Graphs of the distribution frequency of delta (0.5–4 Hz) density of NREM sleep were assessed by one-way ANOVA followed by Bonferroni tests. In all cases, *p* < 0.05 was considered to be statistically significant.

## Results

### Dexmedetomidine Reduced Spontaneous Locomotor Activity in Mice During the Dark Phase

To investigate the sedative effects of orally delivered dexmedetomidine, dexmedetomidine at a dose of 25, 50, 100, or 200 μg/kg was administered (i.g.) at 21:00, and LMA was recorded continuously for 24 h from 19:00. The circadian rhythm of mice means that they spend most of their time sleeping and with less LMA during the light phase. So it is more difficult to assess the effects of drugs on LMA in the light phase than in the dark phase. Therefore, the experiments were performed during the dark phase when animals were active.

**FIGURE 1:**
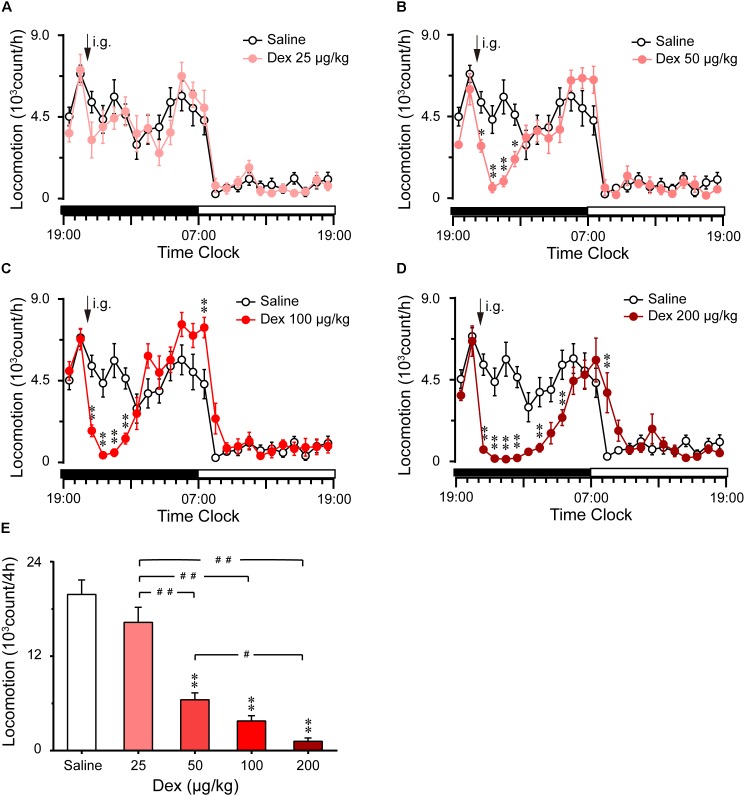
Effects of oral delivery dexmedetomidine on LMA in mice during the dark phase. **(A–D)** Time course changes in LMA following saline (open circle) or dexmedetomidine (25, 50, 100, and 200 μg/kg, i.g.; closed red circle) administration during the dark phase. Each circle represents the hourly mean amount of LMA. The horizontal filled and open bars on the *X*-axes indicate the 12-h dark and 12-h light periods, respectively. Values are means ± SEM (*n* = 8), ^∗^*P* < 0.05 and^∗∗^*P* < 0.01 indicate significant differences compared with the saline control as assessed by two-way repeated measures ANOVA followed by Bonferroni testing. **(E)** Total counts of LMA during the 4-h period following saline or dexmedetomidine (25, 50, 100, and 200 μg/kg, i.g.). Values are means ± SEM (*n* = 8). ^∗∗^*P* < 0.01 indicates a significant difference compared with the control group, *^##^P* < 0.01 indicates a significant difference between the different doses of dexmedetomidine as assessed by one-way ANOVA followed by Bonferroni tests.

As shown in Figures 1B–D, time course changes revealed that dexmedetomidine at 50, 100, and 200 μg/kg significantly decreased LMA in mice during the night phase, which lasted for 2 (*F*_1,158_ = 17.45, *P* < 0.01), 4 (*F*_1,158_ = 8.81, *P* < 0.01), and 8 (*F*_1,158_ = 111.31, *P* < 0.01) hours, respectively. However, dexmedetomidine at 25 μg/kg did not differ significantly from the control group with respect to the LMA cycle (Figure 1A). When dexmedetomidine was increased to 50 μg/kg, LMA was reduced by 87% (*P* < 0.01) and 84% (*P* < 0.01) compared with control group during the second and third hour after administration (Figure 1B). Dexmedetomidine at 100 μg/kg significantly decreased LMA during the first, second, third, and fourth hours by 68, 92, 91, and 73%, respectively compared with the control group. However, LMA rebounded significantly during the first hour of the light period, increasing 1.7-fold (*P* < 0.01) compared with the control group. Despite this, there was no further disruption of LMA architecture during the subsequent period (Figure 1C). In addition, dexmedetomidine at the highest dose of 200 μg/kg decreased LMA for 8 consecutive hours, with significant differences found at the first, second, third, fourth, sixth, and eighth hour and rebounded significantly at the second hour of the light period (Figure 1D).

The total counts of LMA during the 4 h following administration of dexmedetomidine are summarized in Figure 1E. Dexmedetomidine at 50, 100, and 200 μg/kg decreased the total counts of LMA by 67% (*P* < 0.01), 77% (*P* < 0.01), and 82% (*P* < 0.01), respectively, during the 4-h period, compared with the control group. However, dexmedetomidine at 25 μg/kg did not affect the cumulative amount of LMA when measured for 4 h after administration (Figure 1E). These results clearly indicate that dexmedetomidine decreases LMA in a dose-dependent manner.

### Dexmedetomidine Increased NREM Sleep and Decreased Wakefulness in Mice During the Dark Phase

**FIGURE 2:**
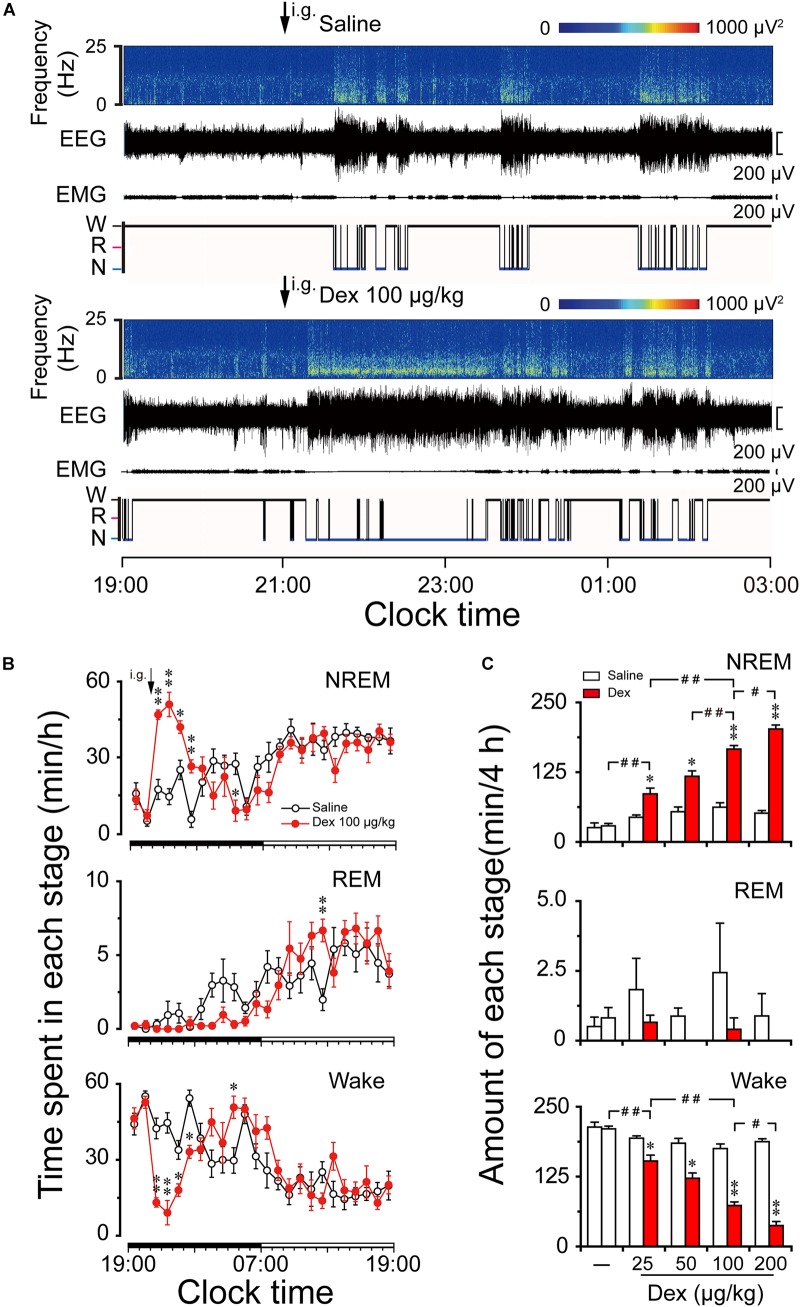
Effects of dexmedetomidine on sleep-wake profiles in mice during the dark phase. **(A)** Typical examples of compressed spectral array (0–25 Hz) EEG, EMG, and hypnograms over 8 h (19:00–03:00) following saline (upper panel) or dexmedetomidine 100 μg/kg (lower panel) administration. **(B)** Time course changes in NREM sleep, REM sleep, and wakefulness following saline (open circle) and dexmedetomidine (100 μg/kg, i.g.; closed red circle) administration during the dark phase. Each circle represents the hourly mean amount of each stage. The horizontal filled and open bars on the *X*-axes indicate the 12-h dark and 12-h light periods, respectively. Values are means ± SEM (*n* = 6); *^∗^P* < 0.05 and *^∗∗^P* < 0.01 indicate significant differences compared with their own control as assessed by two-way repeated measures ANOVA followed by Bonferroni tests. **(C)** Dose-response effects on total time spent in NREM sleep, REM sleep, and wakefulness for 4 h after administration of saline and dexmedetomidine in mice. Open and red filled bars show the profiles of saline and dexmedetomidine treatment, respectively. Values are the means ± SEM (*n* = 6). *^∗^P <* 0.05 and *^∗∗^P <* 0.01 indicate significant differences compared with their own control as assessed by two-tailed paired Student’s *t-*test. *^#^P* < 0.05 and *^##^P* < 0.01 indicate significant differences between saline and different doses of dexmedetomidine as assessed by one-way ANOVA followed by Bonferroni tests.

To investigate the hypnotic effects of oral delivery dexmedetomidine during the dark phase, EEG/EMG were recorded for 2 consecutive days. Typical examples of a compressed spectral array (0–25 Hz) EEG, polygraphic recording, and corresponding hypnograms from a mouse given saline or 100 μg/kg dexmedetomidine are shown in Figure 2A. Mice treated with dexmedetomidine (100 μg/kg) quickly went to the sleep state during which EMG disappeared and they spent more time in NREM sleep compared with their own control.

As shown in Figure 2B, time course changes revealed that dexmedetomidine at 100 μg/kg significantly increased NREM sleep (*F*_1,118_ = 11.24, *P* < 0.01) for 4 h following administration, which is consistent with a reduction in wakefulness (*F*_1,118_ = 6.05, *P* < 0.05) during the same period compared with their own control. Dexmedetomidine at 100 μg/kg increased the hourly NREM sleep time by 2.7- (*P* < 0.01), 3.5- (*P* < 0.01), 1.7- (*P* < 0.05), and 4.8- (*P* < 0.01) fold relative to saline control during the first, second, third, and fourth hour after administration, respectively. The duration of wakefulness was decreased at the same time by 69% (*P* < 0.01), 80% (*P* < 0.01), 47% (*P* < 0.05), and 39% (*P* < 0.01) during the first, second, third and fourth hour after administration, respectively. However, wakefulness rebounded on the eighth hour after administration, which is consistent with a reduction in NREM sleep during the same time. Although time course changes on REM sleep failed to show a significant decrease during the dark phase, REM sleep rebounded 3.4-fold relative to saline control during the 16th hour after administration (*P* < 0.05). There was no further disruption of sleep architecture.

The total time spent in NREM sleep, REM sleep, and wakefulness were measured for 4 h after dexmedetomidine administration because the time course data revealed that 100 μg/kg dexmedetomidine increased NREM sleep for this duration. Dexmedetomidine dose-dependently increased NREM sleep (*F*_4,28_ = 74.22, *P* < 0.01) and reduced wakefulness (*F*_4,28_ = 72.88, *P* < 0.01) (Figure 2C). Dexmedetomidine at 25, 50, 100, and 200 μg/kg increased the total duration of NREM sleep 1.9-, 2.3-, 2.7-, and 3.9-fold, respectively, which was consistent with the reduction in wakefulness by 21, 39, 58, and 78%, respectively, compared with their own control in each group. Due to the small amount of REM sleep in mice during the early dark phase, there was no significant difference in REM sleep within 4 h after dexmedetomidine administration at any dose. Therefore, it reveals that orally delivered dexmedetomidine increases NREM sleep and decreases wakefulness in a dose-dependent manner.

### Dexmedetomidine Shortened Sleep Latency, Altered Sleep–Wake Architecture and EEG Power Density During the Dark Phase in Mice

**FIGURE 3:**
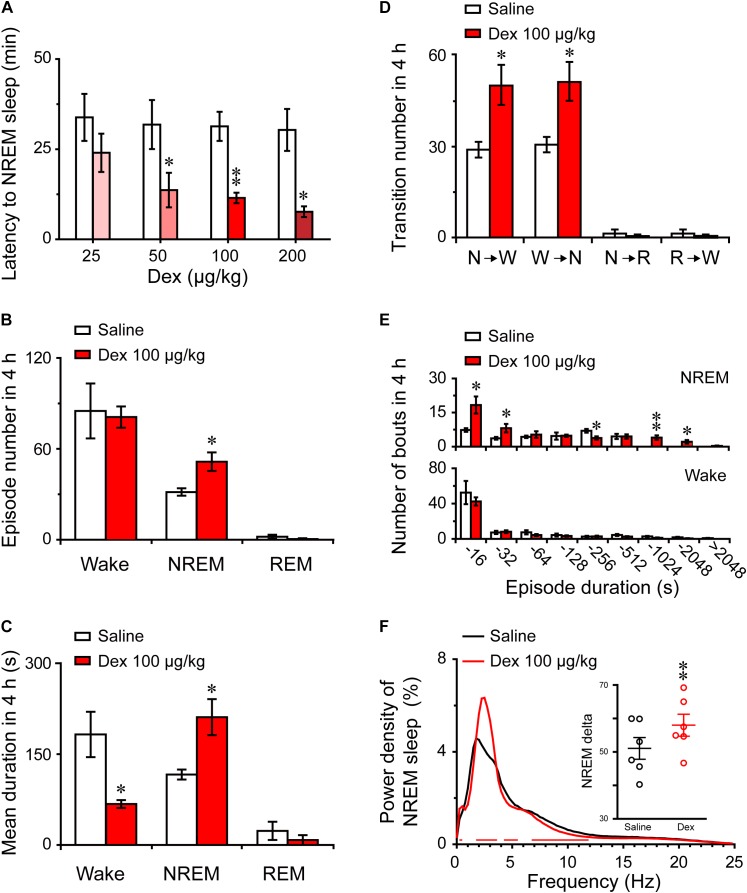
Changes in sleep latency, architecture, and EEG power density of NREM sleep produced by administration of dexmedetomidine. **(A)** Effect of different doses of dexmedetomidine on NREM sleep latency. Values are mean ± SEM (*n* = 6). *^∗^P* < 0.05 and *^∗∗^P* < 0.01 indicate significant differences assessed by two-tailed paired Student’s *t*-test. **(B)** Total episode number, **(C)** mean duration, **(D)** stage transition, and **(E)** number of NREM sleep and wakefulness bouts during the first 4 h following administration of dexmedetomidine 100 μg/kg. Values are mean ± SEM (*n* = 6). *^∗^P* < 0.05 and *^∗∗^P* < 0.01 indicate significant differences when using two-tailed unpaired Student’s *t*-test. **(F)** EEG power density curves of NREM sleep and quantitative changes in power for delta (0.5–4.0 Hz) frequency bands (insert) during the 4 h period after saline and dexmedetomidine (100 μg/kg; i.g.) administration. Red horizontal bars indicate location of a statistically significant difference (*P <* 0.05, two-tailed paired *t*-test). *Y*-axes (insert) indicate the percentage of delta frequency on the EEG power density of NREM sleep. Data (quantitative of delta frequency) were standardized and expressed as the percentage of the mean delta power of NREM sleep. Values are mean ± SEM (*n* = 6). *^∗∗^P* < 0.01 indicates significant differences compared with their own control as assessed by two-tailed paired Student’s *t*-test.

To assess the initiation of the sleep state after treatment, we measured the latencies to NREM sleep, which were defined as the time from saline or dexmedetomidine treatment to the first appearance of a NREM sleep episode that lasted for at least 60 s. As shown in Figure 3A, dexmedetomidine i.g. remarkably shortened NREM sleep latency. The latencies to NREM sleep in mice treated with dexmedetomidine (50, 100, and 200 μg/kg, i.g.) were 13.7 (*P* < 0.05), 11.5 (*P* < 0.01), and 7.7 (*P* < 0.05) min, respectively, which were markedly shorter than the latencies of 31.8, 31.3, and 30.3 min after saline injection. However, 25 μg/kg dexmedetomidine failed to change the latencies to NREM sleep in mince. The short NREM sleep latency following dexmedetomidine (>25 μg/kg, i.g.) clearly indicates that dexmedetomidine accelerates the initiation of NREM sleep.

To better understand the changes in sleep-wake architecture caused by 100 μg/kg dexmedetomidine, we determined the number of episodes and mean duration of wakefulness, NREM sleep, and REM sleep, as well as transitions between the three vigilance stages after dexmedetomidine at a dose of 100 μg/kg. As shown in Figure 3B, dexmedetomidine at 100 μg/kg increased the total number of episodes of NREM sleep 1.6-fold (*P* < 0.05), but there was no significant difference in the number of episodes of wakefulness and REM sleep. In addition, the mean duration of NREM sleep increased by 81% with a concomitant 64% decrease in wakefulness (Figure 3C, *P <* 0.05). As shown in Figure 3D, dexmedetomidine (100 μg/kg) increased the number of state transitions from NREM sleep to wakefulness and wakefulness to NREM sleep (*P* < 0.05) during the 4 h following administration. Neither a change in the number of transitions from NREM sleep to REM sleep nor in that from REM sleep to wakefulness were observed. Distributions of bouts of different durations of NREM sleep and wakefulness are shown in Figure 3E. Dexmedetomidine (100 μg/kg) increased the number of bouts of NREM sleep with durations of 4–32, 128–256, and 512–2048 s. There was no difference in the number of bounds of wakefulness that were observed. These results suggest that dexmedetomidine increases the number of episodes and mean duration of NREM sleep, which extend the overall duration of NREM sleep.

The delta activity (0.5–4 Hz) during NREM sleep is not only a symbol of NREM sleep, but it also reflects the depth of sleep (Anaclet et al., 2014). To better understand the depth of sleep caused by dexmedetomidine, we evaluated the EEG power spectra and compared the power densities of saline and 100 μg/kg dexmedetomidine in mice during NREM sleep. As shown in Figure 3F, the frequency ranges of 1.25 and 2.5–3.5 Hz were increased, with a decrease in the frequency ranges of 4.25–4.5 and 8–11.5 Hz following the administration of 100 μg/kg dexmedetomidine compared with their own control. Then the quantitative changes in power for delta (0.5–4.0 Hz) frequency bands during the 4-h period after saline and dexmedetomidine (100 μg/kg; i.g.) administration were measured. As shown in the insertion part of the diagram in Figure 3F, dexmedetomidine increased the quantitative delta power 1.14-fold (*P* < 0.01) compared with the self-controls. These results suggest that dexmedetomidine increased the duration of NREM sleep and also increased the depth of sleep.

### Dexmedetomidine Increased NREM Sleep, and Decreased REM Sleep and Wakefulness in Mice During the Light Phase

**FIGURE 4:**
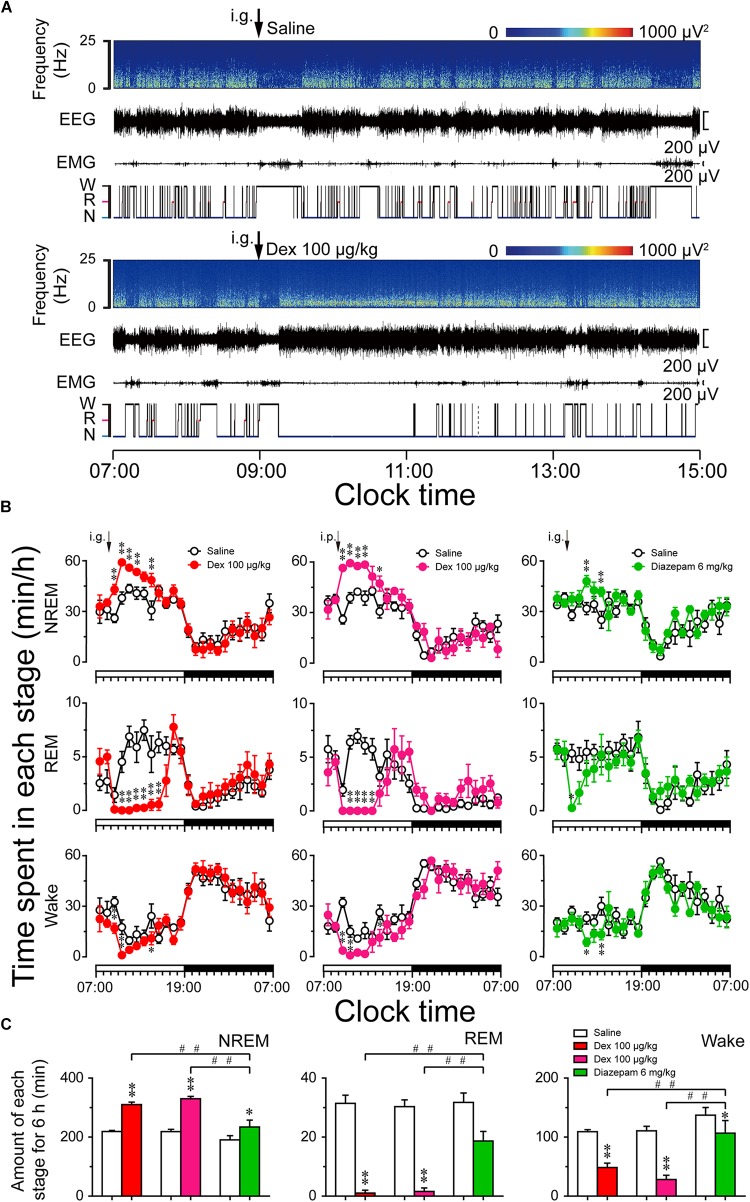
Sleep–wake profiles produced by administration of dexmedetomidine in mice during the light phase. **(A)** Typical examples of compressed spectral array (0–25 Hz) EEG, EMG, and hypnograms over 8 h (07:00–15:00) following saline (upper panel) and dexmedetomidine 100 μg/kg (lower panel) administration. **(B)** Time course changes in NREM sleep, REM sleep, and wakefulness following saline (open circle), dexmedetomidine i.g. (100 μg/kg; closed red circle), dexmedetomidine i.p. (100 μg/kg; closed rose red circle), and diazepam i.g. (6 mg/kg; closed green circle) administration during the light phase. The horizontal open and filled bars on the *X*-axes indicate the 12-h light and 12-h dark periods, respectively. Each circle represents the hourly mean ± SEM of NREM sleep, REM sleep, and wakefulness (*n* = 6). *^∗^P* < 0.05 and *^∗∗^P* < 0.01 indicate significant differences compared with their own control as assessed by two-way repeated measures ANOVA followed by Bonferroni tests. **(C)** Total time spent in NREM sleep, REM sleep, and wakefulness for 6 h after administration. Open, red, rose red, and green filled bars show the profiles of saline, dexmedetomidine (i.g. or i.p.), and diazepam i.g. treatments, respectively. Values are mean ± SEM (*n* = 6). *^∗^P* < 0.05 and *^∗∗^P* < 0.01 indicate significant differences compared with their own control as assessed by two-tailed paired Student’s *t-*test. ^##^*P* < 0.01 indicate significant differences compared dexmedetomidine i.g. with dexmedetomidine i.p. and diazepam i.g. as assessed by one-way ANOVA followed by Bonferroni tests.

The light phase in mice is equivalent to the nighttime sleep stage in humans, so we assessed the effects of dexmedetomidine on sleep-wake profiles during the light period. On day 1, the mice were treated with saline at 09:00 in the early phase of the light period, and the recordings made on that day served as each animal’s own control. The animals were then treated with dexmedetomidine (100 μg/kg, i.g. or i.p.) and diazepam (6 mg/kg, i.g.) 24 h later. Typical examples of compressed spectral array (0–25 Hz) EEG, polygraphic recording, and corresponding hypnograms from a mouse given saline or dexmedetomidine i.g. at a dose of 100 μg/kg are shown in Figure 4A. Mice treated with dexmedetomidine quickly went to into a sleep state with EMG disappearing and more continuous NREM sleep observed compared with each animal’s own control.

As shown in Figure 4B, time course changes revealed that dexmedetomidine (100 μg/kg, i.g.) significantly increased NREM sleep (*F*_1,118_ = 75, *P* < 0.01) and decreased wakefulness (*F*_1,118_ = 21.48, *P* < 0.01) in mice compared with their own control. Dexmedetomidine increased the hourly NREM sleep time 1.66- (*P* < 0.01), 1.56- (*P* < 0.01), 1.29- (*P* < 0.05), 1.31- (*P* < 0.01), and 1.59- (*P* < 0.01) fold relative to saline control during the first, second, third, fourth, and sixth hours after administration, respectively. The enhancement of NREM sleep was concomitant with a decrease in wakefulness during the first, second, and sixth hours after the administration of dexmedetomidine. In addition, dexmedetomidine (100 μg/kg, i.g.) decreased REM sleep for 6 h from the second hour after administration (*F*_1,118_ = 83.52, *P* < 0.01). The effects began within the first hour and lasted for 6 h. Although the route of administration is different, the effect of intraperitoneal administration (i.p.) is almost the same as that of oral delivery. Dexmedetomidine (100 μg/kg, i.p.) increased NREM sleep for 6 h, and this was significant during the first, second, third, fourth, and sixth hours, respectively, compared with their own control (*F*_1,118_ = 54.79, *P* < 0.01). There was no further disruption of sleep architecture during the subsequent period with the two different routes of administration of dexmedetomidine. However, high dose of the classical hypnotic drug diazepam at 6 mg/kg oral delivery only increased NREM sleep by 2 h compared to its own control (*F*_1,118_ = 17.68, *P* < 0.01). There were no time course changes during the dark phase.

To better understand the total time spent in NREM sleep, REM sleep, and wakefulness, each stage was measured for 6 h after dexmedetomidine administration. As shown in Figure 4C, dexmedetomidine (100 μg/kg, i.g.) increased the total duration of NREM sleep 1.4-fold (*P* < 0.01) than saline control, which was consistent with a reduction in wakefulness by 56% (*P* < 0.01) and REM sleep by 97% (*P* < 0.01), respectively. These results suggest that dexmedetomidine increases NREM sleep partially by reducing REM sleep during the light phase. In addition, dexmedetomidine (100 μg/kg, i.p.) and diazepam (6 mg/kg, i.g.) increased the total duration of NREM sleep 1.51-fold (*P* < 0.01) and 1.23-fold (*P* < 0.05) than its own control, respectively. There was no significant difference when the total time spent in NREM sleep following dexmedetomidine i.g. with i.p. was compared. However, both routes of dexmedetomidine administration increased the total amount of NREM sleep significantly more than diazepam, suggesting a strong hypnotic effect of dexmedetomidine.

### Dexmedetomidine Shortened Sleep Latency, Consolidated Sleep Structure, and Increased EEG Power Density of NREM Sleep

**FIGURE 5:**
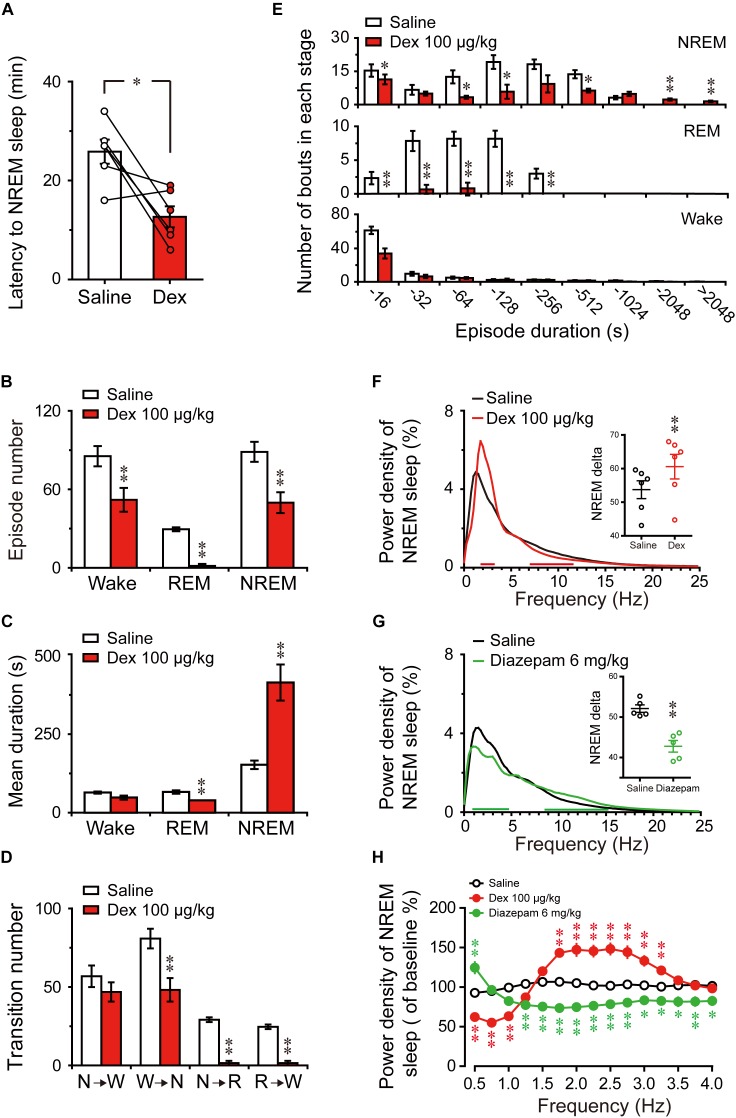
Changes in sleep latency, architecture, and EEG power density of NREM sleep produced by administration of dexmedetomidine during the light phase. **(A)** Effect of dexmedetomidine 100 μg/kg on NREM sleep latency. Values are mean ± SEM (*n* = 6). ^∗^*P* < 0.05 indicates significant differences assessed by two-tailed paired Student’s *t-*test. **(B)** Total episode number, **(C)** mean duration, **(D)** stage transition, and **(E)** number of NREM, REM, wakefulness bouts during the first 6 h following administration of saline or dexmedetomidine 100 μg/kg. Values are mean ± SEM (*n* = 6). ^∗^*P* < 0.05 and ^∗∗^*P* < 0.01 indicate significant differences performed using two-tailed unpaired Student’s *t-*test. **(F,G)** EEG power density curves during NREM sleep and quantitative changes in power for delta (0.5–4.0 Hz) frequency bands (insert) during the 6-h period after saline, dexmedetomidine 100 μg/kg, or diazepam 6 mg/kg administrations. Red or green horizontal bars indicate the location of a statistically significant difference (^∗^*P <* 0.05, two-tailed paired *t*-test). Black, red, and green open circles in the inserted scatter plot represent saline control, administration of dexmedetomidine, and diazepam, respectively. *Y*-axes (insert) indicate the percentage of delta frequency on the EEG power density of NREM sleep. Data were standardized and expressed as the percentage of the mean delta power of NREM sleep. Values are mean ± SEM (*n* = 5, 6). ^∗∗^*P* < 0.01 indicates significant differences compared with their own control as assessed by two-tailed paired Student’s *t-*test. **(H)** The distribution of percentage of delta frequency in NREM sleep over baseline during the 6-h period after saline, dexmedetomidine 100 μg/kg, or diazepam 6 mg/kg. Open and closed red and green circles indicate the profiles of saline, dexmedetomidine, and diazepam treatment, respectively. The value of power spectrum after the first saline administration was defined as baseline. Data were standardized and expressed as percentages of baseline. Values are mean ± SEM (*n* = 5, 6). ^∗^*P* < 0.05 and ^∗∗^*P* < 0.01 indicate significant differences performed by one-way ANOVA followed by Bonferroni tests.

As shown in Figure 5A, dexmedetomidine i.g. remarkably shortened NREM sleep latency during the light phase. The latencies to NREM sleep in mice treated with dexmedetomidine (100 μg/kg) were 13 min, thus markedly shorter than the latency of 26 min after saline administration (*P* < 0.05). As shown in Figure 5B, the total number of episodes of wakefulness, REM sleep, and NREM sleep decreased by 39% (*P* < 0.01), 95% (*P* < 0.01), and 44% (*P* < 0.01), respectively, within 6 h of administration. However, the mean duration of NREM sleep increased 2.7-fold (*P* < 0.01) with a concomitant 90% decrease in REM sleep (*P* < 0.01) (Figure 5C). As mice were asleep during the light phase, there was no difference in the mean duration of wakefulness (Figure 5C). As shown in Figure 5D, dexmedetomidine (100 μg/kg) decreased the number of state transitions from NREM sleep to wakefulness (*P* > 0.05), wakefulness to NREM sleep (*P* < 0.01), NREM sleep to REM sleep (*P* < 0.01), and REM sleep to wakefulness (*P* < 0.01). These findings indicate that dexmedetomidine reduced NREM sleep fragmentation and improved the continuity of NREM sleep by decreasing the transition between each stage.

To better understand the changes in sleep architecture caused by dexmedetomidine (100 μg/kg) during the light phase, the distribution of bouts of each stage was determined as a function of duration of the bout (Figure 5E). Dexmedetomidine (100 μg/kg) decreased the number of bouts of NREM sleep that had durations of 4–16 (*P* < 0.05), 32–64 (*P* < 0.05), 64–128 (*P* < 0.05), and 256–512 s (*P* < 0.05), but increased the number of bouts of NREM sleep that had durations of 1024—2024 (*P* < 0.01) and >2024 s (*P* < 0.01). Simultaneously, the number of bouts of REM sleep that had durations of 16–32 (*P* < 0.01), 32–64 (*P* < 0.01), 64–128 (*P* < 0.01), and 128–256 s (*P* < 0.01) were significantly decreased. However, only the number of bouts of wakefulness of durations of 4–16 s decreased (*P* < 0.01). These results suggest that the increased total time in NREM sleep with dexmedetomidine (100 μg/kg, i.g.) during the light phase was based on the reduction of the quantity of short-term NREM sleep and increase in long-term NREM sleep volume. At the same time, the frequency of awakening and short-term wakefulness, especially for 4–16 s, during the sleep stage was reduced. These occurred along with REM sleep reduction.

Changes in the depth of sleep during the light phase were examined by statically comparing the EEG power density of NREM sleep between dexmedetomidine treatment and saline control. As shown in Figure 5F, frequency ranges of 1.75–3.25 Hz increased and frequency ranges of 7.25–11.75 Hz decreased between dexmedetomidine treatment and their own control. As shown in the insertion part of the diagram in Figure 5F, dexmedetomidine increased the quantitative delta power 1.13-fold (*P* < 0.01) compared with own control. To clarify the effects of the hypnotic diazepam, the delta power of NREM sleep, EEG power spectra, and power densities during NREM sleep for 6 h after oral delivery 6 mg/kg diazepam in mice were compared with their own control. As shown in Figure 5G, the frequency ranges of 0.5 and 1–4.5 Hz decreased while those of 8.75–24.75 Hz increased between diazepam treatment and their own control. Quantitative delta power also decreased by 18% (*P* < 0.01) compared with their own control.

To better understand the distribution changes of wave frequency of delta (0.5–4 Hz) density of NREM sleep, the distribution of percentage of delta frequency in NREM sleep over self-control during the 6 h after saline, dexmedetomidine 100 μg/kg, and diazepam 6 mg/kg were analyzed. As shown in Figure 5H, there was no change in the distribution of delta frequency following saline administration. The frequency ranges of 1.75–3.25 Hz (*P* < 0.01) increased, while ranges of 0.5–1.0 Hz (*P* < 0.01) decreased dexmedetomidine was compared with the saline group. However, diazepam was compared with the saline group, and the frequency ranges of 1.25–4.0 Hz were decreased (*P* < 0.01; *P* < 0.05) and only 0.5 Hz increased (*P* < 0.01). The increased delta density of dexmedetomidine was mainly concentrated in the frequency ranges of 1.75–3.25 Hz, while diazepam reduced most of the delta density spectrum, particularly at the frequency ranges of 1.25–4.0 Hz. In addition, the most common adverse effect of classic hypnotics is residual ‘hangover’ effects, such as drowsiness and impaired psychomotor and cognitive function, and it may persist into the whole day and interfere with work following nighttime administration (Vermeeren, 2004). To clarify the effects of dexmedetomidine on the spectral power of wake during the dark phase following administration, EEG power spectra, and power densities of wake for the first 6 h and the whole 12 h of dark phase were compared with their own control. The results indicate that the administration of dexmedetomidine at 2 h into the light cycle does not affect the wake of mice during the dark phase (Supplementary Figure S1). These results imply that dexmedetomidine does not seem to affect the quality of wake during the dark phase.

### Effects of Dexmedetomidine on c-Fos Expression in the Cerebral Cortex and Sleep–Wake Control Pathway

The number of c-Fos positive neurons in the cortex and sleep-wake control pathway of the brain was counted to investigate the effects of dexmedetomidine. C-Fos protein as a marker for neuronal activity shows changes in different brain regions during spontaneous sleep-wake episodes (Dentico et al., 2009). Here, we administered dexmedetomidine 100 μg/kg at 21:00, at which time mice are spontaneously active and awake. The control group was administered an equal volume of sterile saline and the whole process was operated under 10 lx red light. Animals were then put back into their own cage and perfused after 120 min.

**FIGURE 6:**
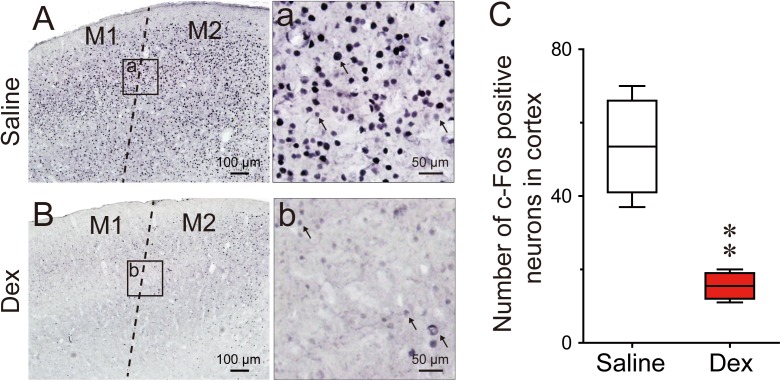
Effects of dexmedetomidine on c-Fos expression in the cerebral cortex during the dark phase. **(A,a,B,b)** Representative photomicrographs of c-Fos expression in the cerebral cortex after saline or dexmedetomidine treatment during the dark phase. **(a,b)** High-magnification views of the square areas marked in **(a,b)** from **(A,B)**; the arrowheads indicate c-Fos positive cells in the cerebral cortex. Scale bars: left panel, 100 μm; right panel, 50 μm. **(C)** Amount of c-Fos positive cells in the square areas (200 × 200 μm) of the cerebral cortex 120 min after treatment with saline or dexmedetomidine 100 μg/kg during the dark phase. Values are expressed as means ± SEM (*n* = 4), *^∗∗^P* < 0.01 indicates significant differences from saline group assessed by two-tailed unpaired Student’s *t-*test.

Previous research has found that c-Fos expression is significantly higher in the cerebral cortex during wakefulness compared to sleep, especially in the prefrontal and frontal, motor, and sensory cortices (Qiu et al., 2014). To determine the effects of dexmedetomidine on c-Fos expression in the cerebral cortex, we selected the motor cortex for comparison, because dexmedetomidine clearly decreases LMA. As shown in Figures 6A,B,a,b, the representative photomicrographs of c-Fos expression in the motor cortices of saline and dexmedetomidine clearly indicate that c-Fos expression in the cortex was very low compared to saline control. Analysis of the number of c-Fos immunoreactive nuclei showed that dexmedetomidine 100 μg/kg decreased the number in the motor cortex by 71% (*P* < 0.01; Figure 6C). These results indicate that dexmedetomidine inhibits the activity of the cerebral cortex, which is consistent with the observations of decreased LMA and increased NREN sleep induced by dexmedetomidine during the dark phase.

**FIGURE 7:**
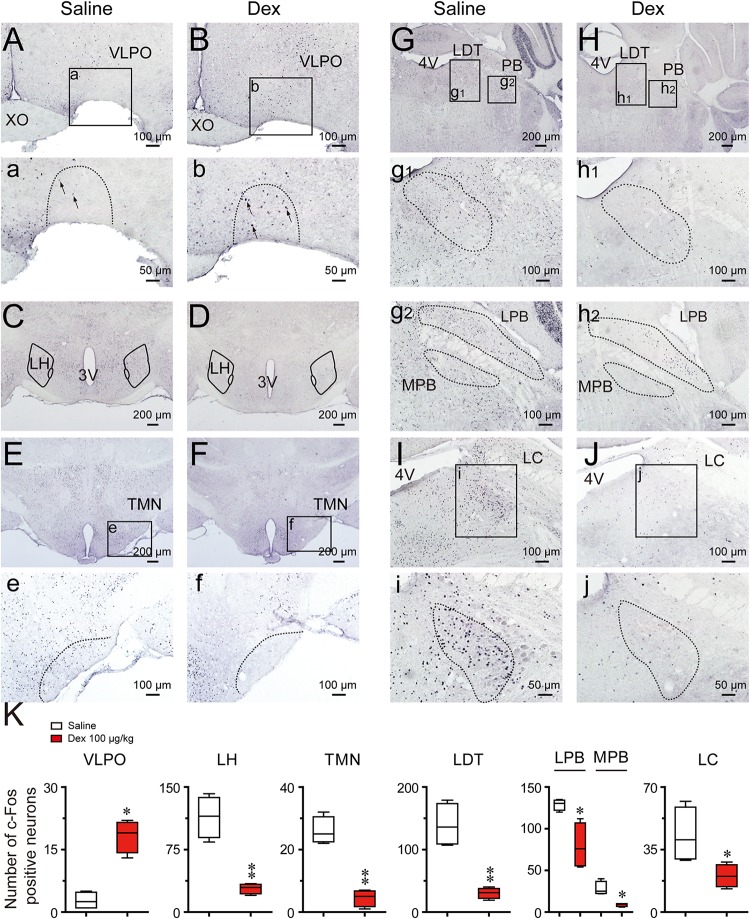
Effects of dexmedetomidine on c-Fos expression in the VLPO and subcortical arousal systems in mice during the dark phase. **(A–J)** Representative photomicrographs of c-Fos expression in the VLPO, LH, TMN, LDT, MPB, LPB, and LC treated saline or dexmedetomidine 100 μg/kg. **(a–j)** High-magnification views of different brain areas treatment with saline or dexmedetomidine. **(K)** Amount of c-Fos positive cells after 120 min following treatment with saline or dexmedetomidine 100 μg/kg during the dark phase. Values are expressed as means ± SEM (*n* = 4), ^∗^*P* < 0.05 and ^∗∗^*P* < 0.01 indicate significant differences between saline and dexmedetomidine treatment group assessed using two-tailed unpaired Student’s *t*-test.

Figure 7 shows representative photomicrographs of c-Fos expression in different brain regions of saline and dexmedetomidine treated mice. As shown in Figures 7A,B,a,b,K, compared with the control group, the administration of dexmedetomidine significantly increased c-Fos expression in the VLPO (*P* < 0.05). Thus, dexmedetomidine exerted hypnotic effects partially by exciting the sleep-promoting nuclear VLPO. However, in contrast with sub-cortical arousal regions such as the LH (*P* < 0.01; Figures 7C,D), TMN (*P* < 0.01; Figures 7E,F,e,f), LDT (*P* < 0.01; Figures 7G,H,g1,h1), LPB and MPB (*P* < 0.05; Figures 7G,H,g2,h2), and LC (*P* < 0.05; Figures 7I,J,i,j), c-Fos expression was significantly lower after the administration of dexmedetomidine compared with the control group (Figure 7K). These subcortical nucleuses are not only wake-initiating but also wake-maintaining components. Results are consistent with the observation that dexmedetomidine significantly decreased wakefulness and increased NREM sleep during the dark phase.

## Discussion

Before dexmedetomidine became readily available around the world, propofol and benzodiazepines were the most frequently used agents for continuous sedation and clinical surgery anesthesia. Since dexmedetomidine was approved by the U.S. Food and Drug Administration in 1999, it has been widely used due to its lack of suppression of respiratory function, protective effect on the nervous system, anesthetic-sparing activity, and attenuation of immunosuppression (Mantz et al., 2011). Both propofol and GABA-receptor agonists have been used to treat insomnia, but they have not been popularized due to respiratory depression, drug resistance, and effect of decreasing sleep depth (NIH, 2004). Whether or not dexmedetomidine can be a drug for treating sleep disorders remains poorly specified. Decreasing LMA is usually considered to reflect sedative effects. In the present study, to verify the sedative effects of orally delivered dexmedetomidine, we initially evaluated the drug by LMA. We found that orally delivered dexmedetomidine dose-dependently decreased LMA during the dark phase, and with a rebound increased in LMA at the later stage. This is consistent with the results of intraperitoneal injection (Seidel et al., 1995).

However, changes in LMA do not directly confirm that dexmedetomidine is increasing sleep. By analyzing EEG/EMG data, we found that the oral delivery of dexmedetomidine dose-dependently increased NREM sleep by improving the mean duration and number of episodes of NREM sleep during the dark phase. Shortening of sleep latency is one of the indicators used to evaluate hypnotic drugs. In the present study, dexmedetomidine reduced the latency to NREM sleep, consistent with the behavior of other hypnotics (Huedo-Medina et al., 2012; Gerashchenko et al., 2017). Fragmented sleep, like short duration sleep bouts and frequent sleep-wake state transitions during sleep are associated with a variety of disorders, including psychiatric disease, memory impairment, and deposition of toxic proteins in cerebrospinal fluid (Wulff et al., 2010; Krause et al., 2017). Our study demonstrates that dexmedetomidine can reduce the quantity of short-term NREM sleep and increase long-term NREM sleep volume through the elimination of the bouts of awakenings during the light phase. This is similar to principal hypnotics (Xu et al., 2014; Manconi et al., 2017; de Zambotti et al., 2018). However, it is worth noting that in healthy mice, sleep fragmentation is not detrimental, which can help mice to raise vigilance and minimize risk of predation during sleep. In present study, we found that dexmedetomidine reduced sleep fragmentation in healthy mice during the light phase, which seems to imply that dexmedetomidine may provide new treatment for sleep-disrupted sleep disorders. To validate this conjecture, researches on animal models of sleep disorders must be conducted.

In addition, these classic hypnotics always decrease depth of sleep, especially the delta activity within NREM sleep (Huedo-Medina et al., 2012; Xu et al., 2014; Gerashchenko et al., 2017). However, dexmedetomidine-induced sleep with increased the delta spectrum and promoted sleep depth. Sleep depth is essential for the brain function maintenance such as eliminating metabolic wastes, promoting learning and memory, cognitive function, and recovery from physical impairments (Xie et al., 2013). And slow wave sleep (deep NREM sleep) disruption increases cerebrospinal fluid amyloid-β levels, an early and necessary step in Alzheimer’s disease pathogenesis (Ju et al., 2017). From this point of view, although dexmedetomidine-induced sleep is different from physiological sleep (increase the delta spectrum), it does not reduce sleep depth compared with traditional hypnotics. In addition, study have found that dexmedetomidine-induced sleep is very similar to sleep rebound after sleep deprivation, both with an increase in delta spectrum (Zhang et al., 2015). In humans, consistent results have found that intravenous injection of dexmedetomidine can promote biomimetic N3 sleep (slow wave sleep) (Akeju et al., 2018).

In this study, dexmedetomidine induced an increase in the high delta frequency range (1.75–3.25 Hz) while a significant decrease in the low delta frequency range (0.5-1 Hz). Although, the delta wave is mainly composed of a wave frequency of 0.5–4 Hz, there is no literature reports the functions of different frequency ranges. In addition, the increase in delta spectrum of NREM sleep during the recovery sleep after sleep deprivation has long been recognized (Zhang et al., 2015), but the effects of sleep deprivation on the distribution of delta wave frequency during the recovery sleep still waiting to study. At present, the understanding of the distribution of delta wave frequency during NREM sleep is also limited. If later studies can find the function of different delta wave frequencies, it will help to further explain the regulation of dexmedetomidine on NREM sleep.

Other research has demonstrated that dexmedetomidine (0.3 mg/kg i.p.) causes sedation and is not a substitute for normal physiologic sleep, with a rebound in both NREM sleep and REM sleep in rats (Garrity et al., 2015). However, whether the animal is in a sleep or anesthetized stage must be distinguished. Intravenous dexmedetomidine at 50 μg/kg or subcutaneous dexmedetomidine at 150 μg/kg can effectively cause loss of the righting reflex (LORR) in rats (Nelson et al., 2003; Hu et al., 2012), and higher than sedative concentrations (400 μg/kg i.p.) were available to cause LORR in the C57BL/6 mice (Gelegen et al., 2014; Zhang et al., 2015). Although the clinical practice guidelines from the American Society of Anesthesiologists classification never refers to sedation or anesthesia as sleep, high doses of dexmedetomidine intraperitoneal injection can put animals in a deeper state than sedation or sleep. The doses given in the present experiment (100 μg/kg) and the route of administration (i.g.) were not sufficient to introduce anesthesia in the animals (Supplementary Figure S2). In addition, clinical trials have also confirmed that low-dose dexmedetomidine can improve postoperative sleep quality (Wu et al., 2016; Lu et al., 2017). In this study, we also found that both intraperitoneal injection and orally delivered dexmedetomidine can both increase NREM sleep for 6 h during the light phase, suggesting that oral delivery dexmedetomidine could offer a good hypnotic effect.

Classic research studies have shown that the neural circuits involved in generating sleep contribute to the loss of wakefulness caused by anesthetic agents and hypnotics (Lu et al., 2008; Hudetz, 2012; Leung et al., 2014). Consistent with this hypothesis, data suggest that dexmedetomidine-induced sleep is caused, in part, by activating sleep-promoting nuclei and inhibiting wake-promoting nuclei (Nelson et al., 2003). It has been proposed that the VLPO may have a key role in sleep-related processes on the basis of the results of unit recordings (Suntsova et al., 2002, 2007), electrophysiological recordings (Gallopin et al., 2000), anatomical tracing (Uschakov et al., 2007; Chung et al., 2017), and c-Fos immunohistochemistry (Sherin et al., 1996; Dentico et al., 2009). In the present study, we also found c-Fos expression increased in the VLPO, and this is consistent with previous research (Nelson et al., 2003). Despite some studies supporting the argument that the LC is the locus of the hypnotic action of dexmedetomidine (Correa-Sales et al., 1992; Chiu et al., 1995), contradictory findings have shown that dexmedetomidine can still induce sedation in mice unable to synthesize NA (Gilsbach et al., 2009; Hu et al., 2012; Sanders and Maze, 2012) or with selective knockdown of α2_A_ adrenergic receptors in the LC (Zhang et al., 2015). In addition, inhibition of LC neurons does not produce sustained sleep (Carter et al., 2010). These results appear to indicate that dexmedetomidine-induced sedative-hypnotic effects may also act on other wake-promoting regions.

Consistent with other research (Nelson et al., 2003; Luo and Leung, 2011), in the present study, dexmedetomidine also decreased c-Fos expression in the TMN. Silencing of histaminergic neurons during wakefulness promotes slow-wave sleep, but not REM sleep (Williams et al., 2014; Fujita et al., 2017); and this is similar to dexmedetomidine-induced NREM sleep. The LH is an important part of the forebrain that contains orexin neurons, melanin-concentrating hormone containing neurons, GABAergic, and glutamatergic neurons (Yamashita and Yamanaka, 2017). These different types of neurons have been identified to be wake-promoting and project heavily into the VLPO, and are thus well-placed to inhibit sleep-promoting neurons (Schone et al., 2014; Venner et al., 2016; Yamashita and Yamanaka, 2017). In the present study, c-Fos expression also decreased in the LH area after dexmedetomidine, and this is consistent with decreasing neuronal activity in this area during sleep (Yamashita and Yamanaka, 2017). According to the “flip-flop switch” hypothesis (Scammell et al., 2017), NREM sleep induced by dexmedetomidine may depend on the inhibition of TMN and LH by VLPO GABAergic neurons, which are excited by dexmedetomidine.

Inconsistent with our results, Garrity et al. (2015) found that dexmedetomidine did not cause significant changes in c-Fos expression in various regions of the hypothalamus after dexmedetomidine (0.1–0.5 mg/kg; i.p.) in rats. However, same results in mice study from Nelson, et al were found that dexmedetomidine (0.4 mg/kg; i.p.) induced a qualitatively similar pattern of c-Fos expression as our results with decrease in the TMN, LH and an increase in the VLPO (Nelson et al., 2003). In addition, another experiment with mice as subject also found c-Fos expression changes in the VLPO, LPO, and MPO during dexmedetomidine-induced sedation (Zhang et al., 2015). As we all known, the c-Fos protein expresses at 30 min but reaches peak at around 90-120 min after stimulation (Zhong et al., 2014; Zhang et al., 2018). In the Garrity’s paper, rats were decapitated 65 min after administration of dexmedetomidine, while mice were decapitated 2 h after administration in Nelson’s work. In our study, we also selected 2 h as the time point. Taking together, the difference in these results may be caused by the different species of the experimental animals, different dose of dexmedetomidine and the time of decapitating of the animals after administration of dexmedetomidine.

In the present study, we explored the effect of dexmedetomidine on c-Fos expression in the parabrachial nucleus (PB). The PB sends glutamatergic projections to a variety of forebrain structures (basal forebrain, LH, and midline thalamus) and cerebral cortex to promote arousal (Kaur et al., 2013). C-Fos immunohistochemical data also reveal that PB neurons are active during passive emergence from isoflurane general anesthesia (Muindi et al., 2016). However, previous studies did not distinguish different regions of PB influence. It has been found that the LPB is necessary for arousal from sleep in response to CO_2_, while the MPB plays an important role in promoting spontaneous waking (Kaur et al., 2013). Therefore, we compared c-Fos expression levels in the LPB and MPB. Consistent with previous research, dexmedetomidine induced NREM sleep along with decrease extinction in the LPB and MPB.

Electrophysiological studies have found that the noradrenergic neurons in the LC are active during wakefulness, less active during NREM sleep, and quiet during REM sleep (Aston-Jones and Bloom, 1981). Based on the evidence that the locus coeruleus as the main site of action for the sedating effects of dexmedetomidine (Chiu et al., 1995), orally delivered dexmedetomidine might be supposed to cause an increase in REM sleep. In contrast to this prediction, our behavioral outcomes show that dexmedetomidine caused a long-lasting elimination of REM sleep during the light phase. However, c-Fos staining results confirm the truth of that dexmedetomidine inhibits the LC neurons activity with decreased c-Fos expression. Although, the current study does not explain the mechanisms by which oral delivered dexmedetomidine caused a significant decrease in REM sleep, lots of works have found that the LDT plays an important role in the induction and maintenance of REM sleep (Verret et al., 2005; Clement et al., 2011; Sakai, 2015; Cox et al., 2016). Cells in the region of the LDT discharge during waking, decrease firing during NREM sleep and increase firing during REM sleep (Boucetta et al., 2014). And selective optogenetic activation of cholinergic neurons in the LDT during NREM sleep could increase the probability of REM sleep and the number of REM sleep episodes but not the duration of REM sleep episodes (Van Dort et al., 2015). Immunohistochemically identified cholinergic neurons in the LDT express c-Fos in the highest numbers in association with REM sleep (Maloney et al., 1999). In the present study, c-Fos expression in the LDT was decreased, which is consistent with decreased REM sleep after dexmedetomidine. However, studies have found the balance between Ach- and NA-mediated neurotransmission in LDT or PPT may play an important role in the regulation of REM sleep (Jones, 2005). And when acetylcholinesterase inhibitors administered into LDT or PPT alone, the wakefulness were happened; but when administered following depletion of the catecholamine by previous treatment of reserpine, REM sleep would be occurred (Jones, 2004). This is consistent with the result of the LC nucleus stopping discharge during REN sleep. In addition, LDT neurons receive projections from LC, and the cholinergic neurons with α2_A_-adrenergic receptors represented approximately one-half of the LDT ChAT^+^ neurons (Hou et al., 2002), indicating that part of cholinergic neurons in LDT are under an inhibitory influence of NA from the LC. Therefore, the occurrence of REM sleep needs to be based on the reduction excitability of the LC, and relative excitement of the LDT. Taking together, decreased in REM sleep by dexmedetomidine may take place through the inhibition not only the excitability of the LC, but also the REM-ON neuronal activity in the LDT by α2_A_R.

Contrary to dexmedetomidine, studies have found wake-promoting agents such as modafini, caffeine and amphetamine can increase c-Fos expression in the TMN and orexin neurons in the LH (Scammell et al., 2000; Deurveilher et al., 2006; Rotllant et al., 2010). Among them, caffeine and amphetamine can also increase c-Fos expression in the LC (Bennett and Semba, 1998; Rotllant et al., 2010), while, functional MRI found modafinil can improve the high-phasic activity of humans’ locus coeruleus (Minzenberg et al., 2008). In addition, those drugs can all increase c-Fos expression in the ventral tegmental area (VTA), the dorsal raphe nucleus, and cerebral cortex. However, the present study showed that the above brain regions and cortex excited by the wake-promoting drugs were inhibited by dexmedetomidine. Equally, electrophysiological study found that the VLPO excited by dexmedetomidine was also inhibited by modafinil (Gallopin et al., 2004).

Clinical research and systematic reviews have shown that dexmedetomidine can improve postoperative sleep quality (Lu et al., 2017). Recently, a clinical pilot study found that dexmedetomidine promoted N3 sleep in a dose-dependent manner and did not impair performance on a psychomotor vigilance test on the next day (Akeju et al., 2018). However, the route of administration was via continuous intravenous pump and a detailed analysis of changes in the sleep architecture was not provided. In the present study, we also found that the oral delivery of dexmedetomidine can increase the amount of NREM sleep time, shorten sleep latency, stabilize NREM sleep structure, and increase the delta power of NREM sleep. These effects are not solely based solely on the suppression of the LC but also on the suppression of other wake-promoting nuclei, such as TMN, LH, PB, and LDT.

## Conclusion

In conclusion, our results indicate that the oral delivery of dexmedetomidine has sedative and hypnotic effects, and it dose-dependently promotes NREM sleep in mice. These effects may be attributed to the excitation of sleep-promoting nuclei and inhibition of wake-promoting nuclei.

## Author Contributions

Z-XF and HD designed and performed the experiments, analyzed the data, and wrote the paper. W-MQ and WZ designed the experiments, analyzed the data, and wrote the paper.

## Conflict of Interest Statement

The authors declare that the research was conducted in the absence of any commercial or financial relationships that could be construed as a potential conflict of interest.

## Supplementary Material

Figure S1 Changes in EEG power density of wake produced by administration of dexmedetomidine during the light phase. **(A,B)** EEG power density curves during wake and quantitative changes in power for delta (0.5–4.0 Hz) frequency bands (insert) during the 6-h period after saline, dexmedetomidine 100 μg/kg, or diazepam 6 mg/kg administrations. **(C,D)** EEG power density curves during wake and quantitative changes in power for delta (0.5–4.0 Hz) frequency bands (insert) during the 12-h period of dark phase after administrations. **(E,F)** EEG power density curves during wake and quantitative changes in power for delta (0.5–4.0 Hz) frequency bands (insert) during the 6-h period of dark phase after administrations. Red or green horizontal bars indicate the location of a statistically significant difference (^∗^*P* < 0.05, two-tailed paired *t*-test). Black, red, and green open circles in the inserted scatter plot represent saline control, administration of dexmedetomidine, and diazepam, respectively. Y-axes (insert) indicate the percentage of delta frequency on the EEG power density of NREM sleep. Data were standardized and expressed as the percentage of the mean delta power of wake. Values are mean ± SEM (*n* = 5, 6). ^∗∗^*P* < 0.01 indicates significant differences compared with their own control as assessed by two-tailed paired Student’s *t*-test.

Figure S2 External stimulation can wake up the mouse. Typical examples of raw EEG/EMG and following dexmedetomidine 100 μg/kg (upper panel) or dexmedetomidine 200 μg/kg (lower panel) administration in a mouse during the dark phase. (The initial screenshot of NREM sleep transition to wake stage from a mouse; *n* = 6).
