# Supplementary figures and images for: Oral Delivered Dexmedetomidine Promotes and Consolidates Non-rapid Eye Movement Sleep via Sleep–Wake Regulation Systems in Mice
Source: Front Pharmacol. 2018 Dec 5;9:1196. doi: 10.3389/fphar.2018.01196 (PMC6290063; doi:10.3389/fphar.2018.01196)

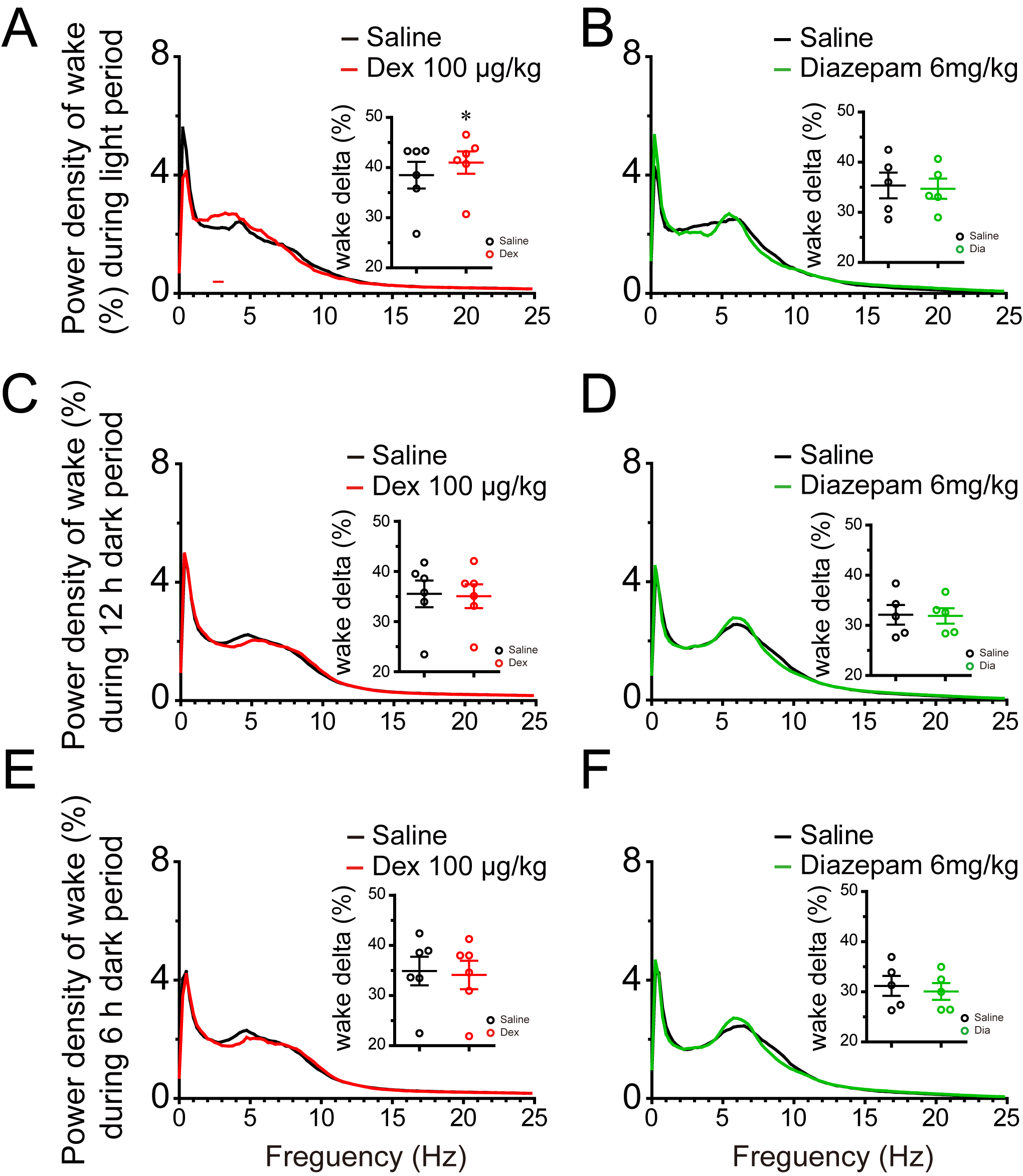

Supplement: Figure S1 — Changes in EEG power density of wake produced by administration of dexmedetomidine during the light phase. (A,B) EEG power density curves during wake and quantitative changes in power for delta (0.5–4.0 Hz) frequency bands (insert) during the 6-h period after saline, dexmedetomidine 100 μg/kg, or diazepam 6 mg/kg administrations. (C,D) EEG power density curves during wake and quantitative changes in power for delta (0.5–4.0 Hz) frequency bands (insert) during the 12-h period of dark phase after administrations. (E,F) EEG power density curves during wake and quantitative changes in power for delta (0.5–4.0 Hz) frequency bands (insert) during the 6-h period of dark phase after administrations. Red or green horizontal bars indicate the location of a statistically significant difference (∗P < 0.05, two-tailed paired t-test). Black, red, and green open circles in the inserted scatter plot represent saline control, administration of dexmedetomidine, and diazepam, respectively. Y-axes (insert) indicate the percentage of delta frequency on the EEG power density of NREM sleep. Data were standardized and expressed as the percentage of the mean delta power of wake. Values are mean ± SEM (n = 5, 6). ∗∗P < 0.01 indicates significant differences compared with their own control as assessed by two-tailed paired Student’s t-test. [file Image_1.TIF]

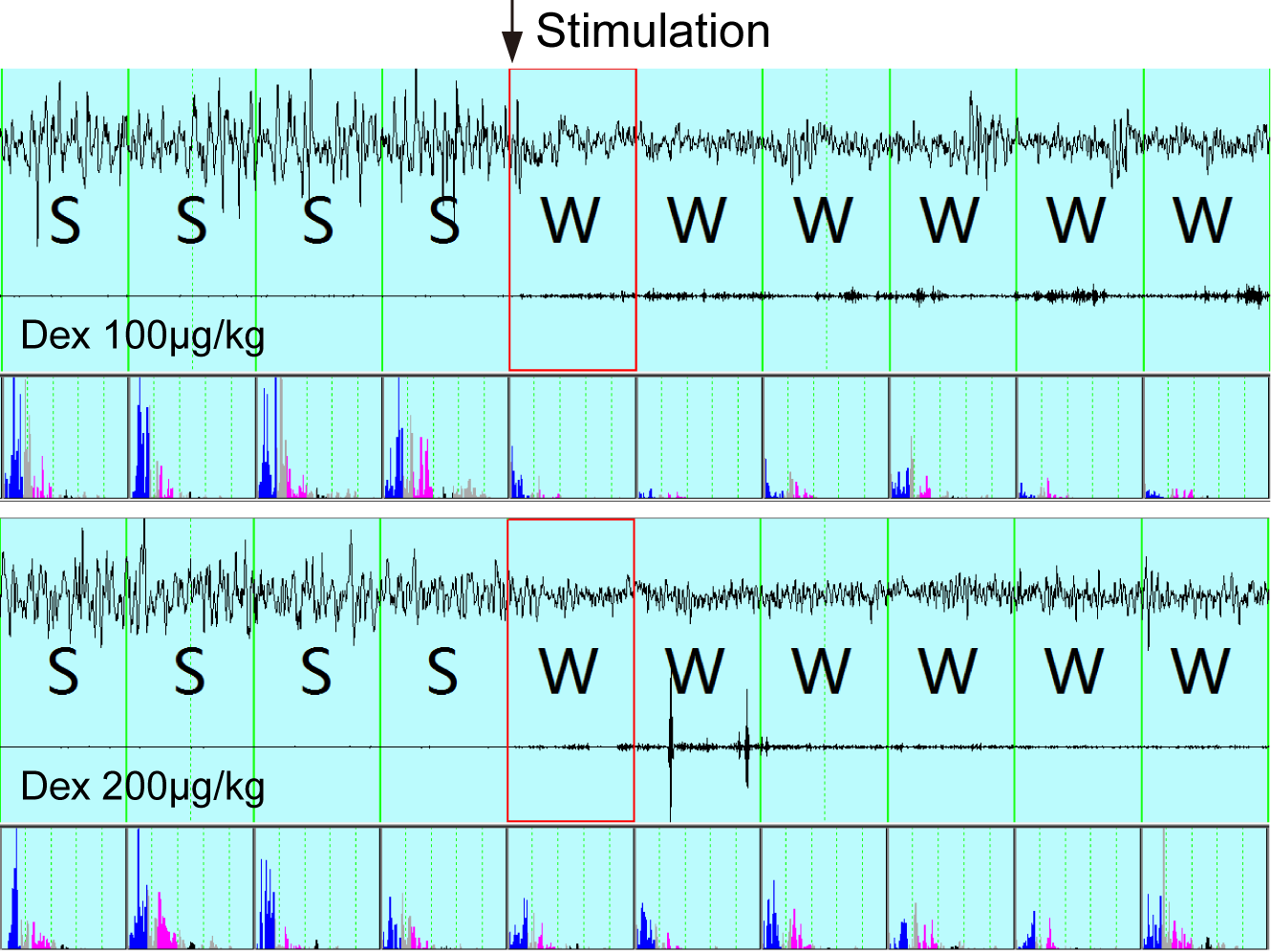

Supplement: Figure S2 — External stimulation can wake up the mouse. Typical examples of raw EEG/EMG and following dexmedetomidine 100 μg/kg (upper panel) or dexmedetomidine 200 μg/kg (lower panel) administration in a mouse during the dark phase. (The initial screenshot of NREM sleep transition to wake stage from a mouse; n = 6). [file Image_2.TIF]
